# Troponin as ischemic biomarker is related with all three echocardiographic risk factors for sudden death in hypertrophic cardiomyopathy (ESC Guidelines 2014)

**DOI:** 10.1186/s12947-017-0115-6

**Published:** 2017-09-13

**Authors:** Rafał Hładij, Renata Rajtar-Salwa, Paweł Petkow Dimitrow

**Affiliations:** 1 2nd Department of Cardiology, Jagiellonian University Medical College, Kraków, Poland; 2 II Klinika Kardiologii CMUJ, ul. Kopernika 17, 31-501 Kraków, Poland

**Keywords:** Hypertrophic cardiomyopathy, Troponin, Echocardiography

## Abstract

**Background:**

Sudden cardiac death (SCD) risk stratification is the most important preventive action in patients with hypertrophic cardiomyopathy (HCM). The identification of the ischemia biomarker high sensitive troponin I (hs-TnI) role for this arrhythmic disease may provide additional information for SCD risk stratification. The aim of the study was to compare echocardiographic parameters (prognostic for risk stratification of SCD in HCM) among two subgroups of HCM patients: with elevated hs-TnI versus non-elevated hs-TnI level.

**Methods:**

In 51 HCM patients (mean age 39 ± 8 years, 31 males and 20 females) an echocardiographic examination, including the stimulating maneuvers to provoke maximized LVOT gradient, was performed. The hs-TnI was measured 24 h later.

**Results:**

By comparing two subgroups of patients, 26 members with hs-TnI positive versus 25 with hs-TnI negative, the study showed that the values of all three parameters were greater: provocable left ventricular outflow tract gradient (LVOTG) – 49.1 ± 45.9 vs 25.5 ± 24.8 mmHg, *p* = 0.019; left atrial diameter – 50.1 ± 9.6 vs 43.9 ± 9.8 mmHg, *p* = 0.041; maximal LV thickness – 22.1 ± 5.3 vs 19.9 ± 34 mm, *p* = 0.029.

**Conclusion:**

The increased value of all three echocardiographic parameters used as risk factors for SCD (ESC Guidelines) is related to the elevated level of hs-TnI in HCM. Due to the high LVOTG – great hs-TnI relationship, exercise stress, both diagnostic and even rehabilitation/training, should be monitored by biomarker control.

## Background

Among risk factors of SCD in HCM in current ESC Guidelines (1), three echocardiographic parameters are included. These echocardiographic parameters are precise due to unequivocal, accurate measurable, in current moment of risk stratification using risk calculator [1]. This calculator for sudden cardiac death risk does not include any biomarker. Calculator also did not include either the ischemic biomarker or signs and symptoms for myocardial ischemia. However, ischemic response to stress revealed by echocardiographic methods becomes more and more important [2, 3].

Very recently, a relationship between nonsustained ventricular tachycardia (ECG-Holter risk factor) and elevated troponin level has been reported [4].

High-sensitive troponin I (hs-TnI) is aprecise biomarker for the detection of myocardial ischemia. In previous HCM studies, the measurements of hs-Tn were performed only at a resting (without stress) echocardiography and not timely synchronized with maneuvers to provoke LVOTG by natural stimuli reflecting daily common physical activities for patients [5–8].

The aim of this study was compare to three echocardiographic parameters (risk factors for SCD: provocable LVOTG, left atrial diameter, maximal LV thickness at end diastole) between subgroups divided by the border value of h-s TnI concentrations in HCM patients. The cut-off value 19,5 ng/l was used according to producer instruction (bioMerieux VIDAS® High sensitive Troponin I). The 99th percentile of a presumably healthy population, the recommended cut-off has been defined at 19,5 ng/L [9].

We have compared echocardiographic parameters between subgroup patients with elevated hs-TnI level versus subgroup patients with non-elevated hs-TnI level.

## Methods

Consecutive patients with HCM were recruited to the study. All patients fulfilled conventional diagnostic criteria for HCM. The exclusion criteria were as follows: ST-segment or non-ST-segment elevation myocardial infarction (recent or previous), significant coronary stenosis on coronary angiography, or renal failure.

The final sample included 51 patients with HCM (mean ± SD age, 39 ± 8 years; 30 men and 21 women). Coronary angiography was performed (due to indications) in 11 patients (result was normal or insignificant coronary artery stenosis <50%).

The patients on current pharmacotherapy were examined in the following 24-h cycle as follows: 8 a.m. echocardiography with LVOTG provocation by natural stimuli (orthostatic test and Valsalva test [1, 10–12]- provocable LVOTG is more preference than resting in SCD calculator [1]) day phase physical activity with probably episodes of provocable LVOTG (unmeasurable), night phase period as a potential time for the rise of troponin, which level has been measured after night at 8 a.m. So far, no studies have used the following protocol. The patients has been asked to performed your common day physical activity and nocturnal resting. This protocol seems to be reasonable because hs-TnI levels may be related to the dynamic and labile nature of LVOTG with fluctuating peaks during the day time (provoked LVOTG as a potential cause for myocardial ischemia). During the first stage of our study we did not use upright exercise stress test to provoke LVOTG [12] because previous study had reported exercise-related Tn release [13].

Methodology of measurement of echocardiographic parameters has been based on ESC Guidelines [1]: As increased ventricular wall thickness can be found at any location, the presence, distribution and severity of hypertrophy should be documented using a standardized protocol for cross-sectional imaging from several projections. Correct orientation and beam alignment along orthogonal planes are essential to avoid oblique sections and over-estimation of wall thickness. Measurements of LV wall thickness should be performed at end-diastole, preferably in short-axis views. The most relevant parameter is the maximum LV wall thickness at any level [1]. Accordingly to ESC Guidelines [1] and previous Italian publication [14] we use anteroposterior LA diameter.

The workflow of echocardiographic examination has been as follows:

- standard echo examination in resting condition in supine position (the last stage of examination was LVOTG measurement using 5-chamber apical window).
- immediately, Valsalva maneuver was perform in supine position with LVOTG measurement
- patients were asked to stand up with their left hand on their head
- LVOTG was measured in 5-chamber apical when patient was in standing position

The study protocol was approved by the local institutional review board (Komisja Bioetyki Jagiellonian University KBET/119/B/2017). Informed consent was obtained from each participant.

### Statistical analysis

Normally distributed continuous variables were presented as mean ± SD. Differences between two groups were assessed using independent t test. Correlations between hs-TnI levels and echocardiographic parameters were assessed using the Pearson correlation coefficient. A *P* value of less than 0.05 was considered significant.

## Results

The characteristics of patients are displayed in Table 1.

**Table 1:** Baseline characteristics of the patients

NYHA class	2.3 ± 0.7
CCS class	1.5 ± 0.5
Syncope	13
nsVT in Holter monitoring	16
Atrial fibrillation	9
Sudden death in family history	11
Creatinine, μg/l	88.2 ± 13.5
Systolic blood pressure mmHg at rest	129 ± 10
Diastolic blood pressure mmHg at rest	69 ± 8

Hs-TnI was detected in all HCM patients and patients with an abnormal level > 19,5 ng/l were defined as positive troponin subgroup.

By comparing the hs-TnI positive versus negative subgroups, the values of all three parameters were greater: left atrial diameter; maximal LV thickness, provocable LVOTG. Also, resting LVOTG was greater in hs-TnI positive subgroup (Table 2).

**Table 2:** Comparison between hs-TnI negative and positive subgroup

	Trop negative	Trop positive	
Parameters from echocardiography non-used as risk factors for SCD in calculator			
LV end diastolic diameter (mm)	39.3 ± 6.6	42.1 ± 5.5	NS
Resting EF (%)	60.1 ± 11.7	62.6 ± 10.5	NS
Resting LVOTG (mmHg)	13.1 ± 12.7	33.9 ± 30	*P* = 0.021
Parameters from echocardiography used as risk factors for SCD in calculator			
Provocable LVOTG (mmHg)	25.5 ± 24.8	49.1 ± 45.9	*P* = 0.019
Left atrial diameter (mm)	43.9 ± 9.8	50.1 ± 9.6	*P* = 0.041
Maximal LV thickness (mm)	19.9 ± 3.4	22.1 ± 5.3	*P* = 0.029

The Pearson correlations were statistically significant between provocable LVOTG and hs-TnI (*r* = 0.39, *p* < 0.05) as well as between resting LVOTG and hs-TnI (*r* = 0.37 p < 0.05).

## Discussion

Best to our knowledge, current study is the first ever report about relationship between elevated hs-TnI and all three echocardiographic risk factors (measurements was timely synchronized) for sudden death in hypertrophic cardiomyopathy (ESC Guidelines 2014).

The impact of stress echocardiography in HCM is limited by the lack of standardization and outcome data. ECS guidelines recommend stress echocardiography solely for the evaluation of LVOT [1]. However, large-scale registry data show that SE positivity for ischemic criteria (such as new wall motion abnormalities and coronary flow velocity reserve), rather than inducible gradients predict adverse outcome in HCM [2]. In a large study [2], mortality was predicted using criteria for detecting ischemia on stress echocardiography. The authors concluded that stress echocardiography has an important prognostic role in patients with HCM, with ischemic endpoints showing a greater predictive accuracy than hemodynamic endpoints [2].

There is no unequivocal data regarding the hemodynamic mechanism for myocardial ischemia diagnosed with hs-TnI release in HCM. We suspect that elevated hs-TnI levels (related to episodes of provocable LVOT) are quite common during the daily activities of patients with HCM (even in pharmacotherapy). Therefore, we investigate the presence of and potential hemodynamic mechanism underlying the increased hs-TnI levels in relation to the findings on resting and provocable LVOTG.

Previously, in experimental studies (resting supine position) ischemia was induced by rapid atrial pacing and beta-receptor stimulation [15, 16]. Formerly, invasive measured marker of myocardial ischemia was lactate metabolism (myocardial lactate extraction indicated the difference between arterial and great cardiac vein lactate contents) [15, 16]. Negative difference was defined as lactate production and preciously indicate myocardial ischemia.

In HCM patients with LVOTG [15], transmural coronary flow reserve was exhausted at a heart rate of 130 beats/min; higher heart rates resulted in more severe metabolic evidence of ischemia (lactate production instead consumption) with all patients experiencing chest pain. At a heart rate of 150, most patients with basal LVOTG demonstrated an actual decline in coronary flow, which correlated with more severe metabolic evidence of ischemia [15]. In a study by Cannon et al. [17], majority patients with HCM with reversible thallium-201 abnormalities during exercise showed metabolic evidence of myocardial ischemia during rapid pacing (myocardial lactate production) associated with increase of LV end diastolic pressure. Beta-receptor stimulation increased LVOTG parallel with lactate production [16] and oppositely surgical reduction of LVOTG was associated with a beneficial shift from lactate production to consumption [18].

In previous study [19] ischemic injury assessed by CK-MB (long-term follow-up was started in 1987/88 years) was related with increasing left atrial diameter. In our study without follow-up, we use more sensitive biomarker and increased TnI level is related with greater left atrial diameter.

Currently, we can use an almost a perfect marker of ischemia, sampled from a peripheral vein i.e. troponin measured by the high-sensitive method.

In a recent review by McCarthy et al. [20], the utility of hs-Tn assessment in arrhythmic disease is only at the initial stage, but it has been proposed as a valuable screening marker for patients with HCM at a high risk of SCD.

Regular training exercise has recently been recommended for selected patients with HCM [21]. In our opinion, based on the association between provocable LVOTG and elevated troponin level in patients with HCM, we suggest that any exercise stress test in HCM patients (performed either for training or diagnostic purposes) should be controlled by troponin level measurements 6 and 12 h after the exercise).

Our study has several limitations. First, the current pharmacological treatment was maintained, and particularly β-blockers were not withdrawn. In our pilot study, we aimed to make the first-ever observation on the correlation between hs-TnI release and timely synchronized findings on echocardiography. Our preliminary study showed that β-blocker withdrawal or specially performed exercise test might not be safe among this group of patients. In future studies, we will attempt to increase the dose and use only one type of a β-blocker to decrease ischemia burden and the risk of troponin release in exercise test. We are going to perform upright exercise with LVOTG measurement at peak and post-exercise in upright position [10, 22–24].

We decided to measure hs-TnI levels only once because our pilot study was conducted in an outpatient setting. The optimal protocol, that is, 48-h profile of troponin measurement with the assessment of troponin with echocardiographic examination every 8 h, would require the in-hospital setting for the study and would be more costly. Moreover, only an outpatient-based study provides an opportunity to assess patients during their common daily physical activities of patients, however daily activities between the patients were not well standardized by for example by questionnaire. The patients were asked to perform their common daily physical activities and nocturnal resting.

## Conclusion

The increased value of all three echocardiographic parameters using as risk factors for sudden death are related to the elevated level of h-s troponin-I in HCM.

### Clinical perspective

These findings suggest that hs-Tn may be useful as an additional biomarker for better risk stratification in HCM. Additionally, we have postulated that every exercise test in HCM should be controlled by the measurement of the troponin level after 8–12 h from the stress test.

## Abbreviations

ESC: European Society of Cardiology
HCM: Hypertrophic cardiomyopathy
Hs-TnI: High sensitivity troponin I
LVOTG: Left ventricular outflow tract gradient
SCD: Sudden cardiac death
